# Association of remnant cholesterol with decreased kidney function or albuminuria: a population-based study in the U.S.

**DOI:** 10.1186/s12944-023-01995-w

**Published:** 2024-01-04

**Authors:** Xuan He, Renfang Zou, Xiaoqiong Du, Kuo Li, Dujuan Sha

**Affiliations:** 1 Department of General Practice, Nanjing University Medical School Affiliated Nanjing Drum Tower Hospital, Nanjing, 210008 China; 2 Nanjing Drum Tower Hospital Clinical College of Nanjing University of Chinese Medicine, Nanjing, 210023 China; 3 Nanjing Drum Tower Hospital Clinical College of Nanjing Medical University, Nanjing, 211166 China

**Keywords:** Remnant cholesterol, Decreased kidney function, Albuminuria, NHANES

## Abstract

**Background:**

Dyslipidemia is frequently exhibited in individuals with chronic kidney disease (CKD). Remnant cholesterol (RC), an emerging novel lipid marker, plays an elusive role in CKD progression. This study sought to investigate the association of RC with decreased kidney function or albuminuria in the general population of U.S.

**Method:**

Data were retrieved from the continuous 2001 to 2018 cycle of the National Health and Nutrition Examination Survey (NHANES). Individuals aged between 18 and 70 years were included. RC was divided into quartiles. Albuminuria was defined by albumin-to-creatinine ratio (ACR) ≥30 mg/g, while reduced kidney function was described as an estimated glomerular filtration rate (eGFR) below 60 ml/min/1.73 m^2^. Using a multivariable regression model, the association of RC with decreased eGFR or albuminuria was examined. The dose‒response relationship between RC and eGFR or ACR was also investigated using a restricted cubic spline (RCS) model.

**Results:**

A total of 1551 (10.98%) participants with impaired renal function or albuminuria were identified. After multivariate adjustment, RC was not significantly associated with kidney function decline or albuminuria (odds ratio (OR) 1.24, 95% confidence interval (95% CI): 0.95, 1.61). However, a significantly inverse correlation was observed between RC and eGFR in a dose‒response manner (β -2.12, 95% CI: -3.04, -1.21). This association remained consistent when stratifying data by gender, age, race, hypertension, diabetes and body mass index (BMI).

**Conclusion:**

A higher RC was significantly correlated with a lower eGFR in the general population. The role of RC in predicting kidney outcomes needed further investigation in prospective studies.

**Supplementary Information:**

The online version contains supplementary material available at 10.1186/s12944-023-01995-w.

## Introduction

The growing prevalence of chronic kidney disease (CKD) has resulted in significant morbidity and mortality worldwide, mostly because of cardiovascular complications [1, 2]. A recent analysis revealed that renal dysfunction accounts for 7.6% of annual cardiovascular disease (CVD) deaths [3]. Intensive treatment of conventional risk factors was less effective at reducing CVD risk in individuals with CKD compared to the general population [4], suggesting that kidney dysfunction independently contributes to cardiovascular events. Therefore, to reduce the risk of CKD and CVD, it is vital to identify and control modifiable risk factors that initiate renal dysfunction.

Dyslipidemia is frequently exhibited in people with CKD and is widely recognized as a CVD risk factor. Apart from dysregulated lipoprotein cholesterol levels, lipid disorders in CKD are characterized by increased levels of triglycerides (TG) [5] and triglyceride-rich lipoproteins (TRLs), which consist of very low-density lipoprotein (VLDL), intermediate-density lipoprotein (IDL) and chylomicron remnants [6]. Results from earlier studies regarding the contribution of lipid abnormalities to the development of CKD have been conflicting. Decreased levels of high-density lipoprotein cholesterol (HDL-C) [7], elevated levels of TG [8] and low-density lipoprotein cholesterol (LDL-C) [9], were associated with an increased risk of CKD. However, Rahman and his colleagues [10] observed no correlation between TG levels and CKD development. Moreover, the chronic renal insufficiency cohort (CRIC) study demonstrated a nonindependent relationship between LDL-C or HDL-C and progression of CKD in patients with CKD stages 2–4 [10]. Remnant cholesterol (RC), which constitutes the cholesterol content of TRLs [11], has garnered increasing interest, as multiple cohort and prospective studies have confirmed its causal role in increased CVD risk independent of traditional lipid parameters [12–14]. RC has been shown to play an elusive role in kidney function deterioration among CKD patients [10, 15, 16]. However, it is unclear how RC and renal function are related in the general population.

To determine whether RC could be a biomarker for CKD risk, this study investigated how RC affects renal function and albuminuria in a representative sample of U.S. individuals.

## Materials and methods

### Data sources

The National Health and Nutrition Examination Survey (NHANES) is a program of population-based studies conducted annually in the U.S. communities. Individuals aged two months and older were selected randomly with a complex sample design. Accessible data included organized questionnaires, physical examinations, and laboratory results. The data were continuously collected and released on a two-year cycle. This study retrieved data from the continuous 2001–2018 cycle.

### Study population

**Fig. 1:**
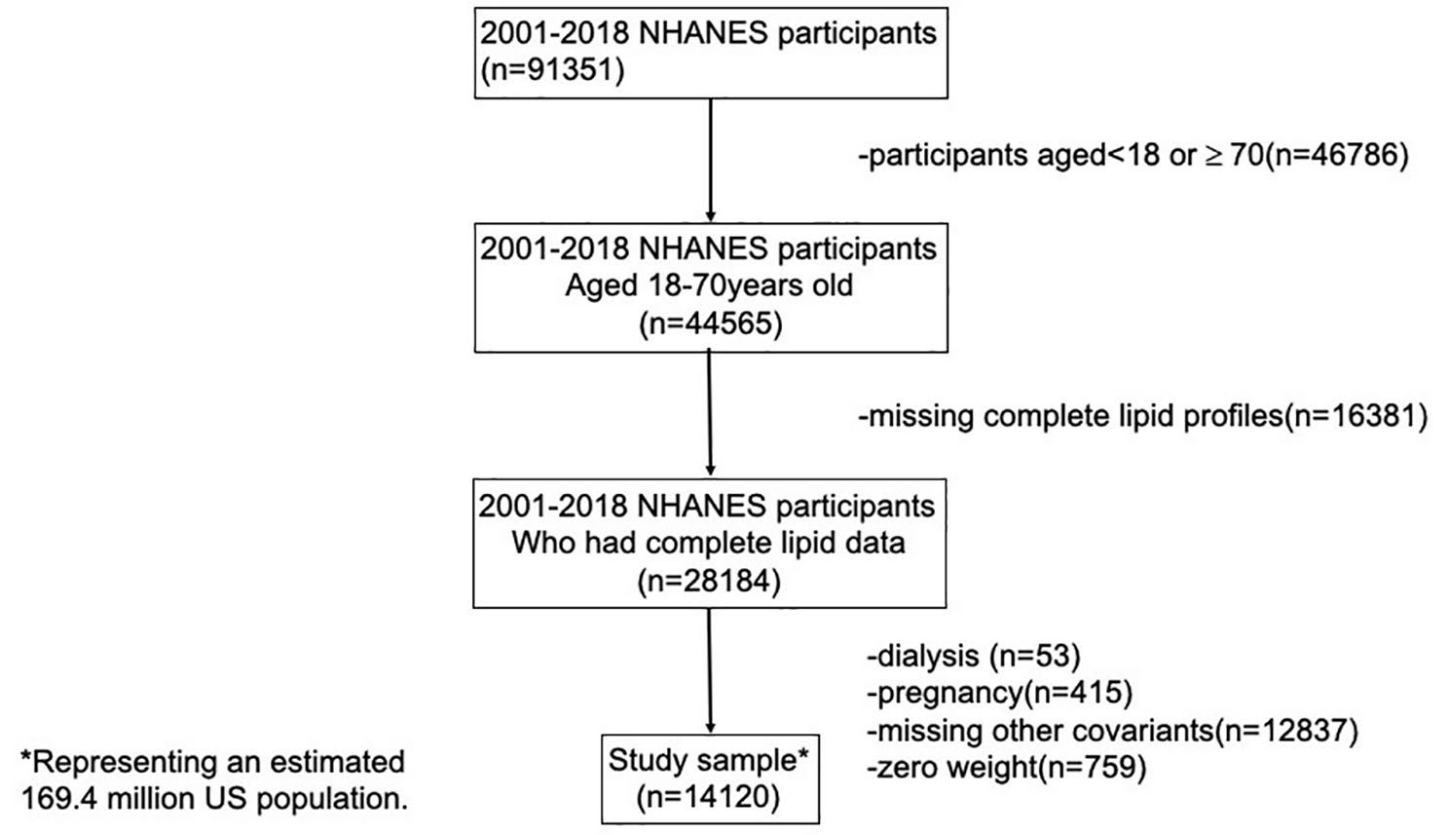
Flow chart of study population

Initially, 91,351 noninstitutionalized subjects were included in our analysis. Participants ≥ 18 and < 70 years of age (n = 44,565) were selected in consideration of the limitations of creatinine-based calculation of estimated glomerular filtration rate (eGFR) [17]. Among the 28,184 individuals with complete lipid profiles, those who reported pregnancy or had a positive pregnancy result upon urine test (n = 415) were excluded, as were those who had undergone dialysis within the previous year (n = 53), because both pregnancy and dialysis may interfere with eGFR levels. Individuals lacking other relevant covariates, such as body mass index (BMI), education, smoking, coronary heart disease, diabetes and hypertension (n = 12,837), were also excluded. 14,210 participants, or an estimated 169.4 million people, were recruited in the final analysis (Fig. 1).

### Lipid measurement

RC was determined by subtracting total cholesterol (TC) from LDL-C and HDL-C. LDL-C levels were calculated from directly measured TC, TG, and HDL-C via the Friedewald equation, which is widely used in clinical practice and research settings. Enzymatic assays were used to measure TC in the laboratory. Either a direct immunoassay method or a heparin-manganese (Mn) precipitation method was used to measure HDL-C levels. To quantify TG, the lipoprotein lipase technique was employed. The NHANES Laboratory/Medical Technologies Procedures Manual provided detailed instructions for the collection and processing of specimens [18].

The quality assurance and quality control (QA/QC) processes in the NHANES comply with the requirements of the 1988 Clinical Laboratory Improvement Act.

### Kidney function evaluation

Reduced kidney function (eGFR < 60 ml/min/1.73 m^2^) and/or albuminuria (albumin-to-creatinine ratio (ACR) ≥30 mg/g) were the main outcome. The secondary outcomes included decreased kidney function, albuminuria, the continuous eGFR and the log-transformed ACR to normalize the residual albuminuria distribution. Serum and urine specimens were taken on the same day. Creatinine concentration was measured via the kinetic rate Jaffe method and recalibrated for the determination of eGFR levels by CKD Epidemiology Collaboration (CKD-EPI) equation [19].

### Covariate assessment

Using standardized questionnaires, sociodemographic characteristics, smoking status, coronary heart disease, antidiabetic and antihypertensive drug use, and lipid-lowering medication use were obtained. Participants were considered underweight to normal if their BMI was below 25 kg/m^2^, overweight if it was between 25 and 30 kg/m^2^, and obese if it was over 30 kg/m^2^ [20]. Smokers were classified into three groups: current smokers who had a history of smoking more than 100 cigarettes and did so occasionally or consistently, former smokers who had smoked more than 100 cigarettes in their lifetime and quit smoking at the examination, and never smokers who had smoked fewer than 100 cigarettes in their lives. Diabetes was diagnosed on the basis of self-reports, antidiabetic drug use, hemoglobin A1C ≥ 6.5%, fasting glucose levels ≥ 7.0 mmol/L, postprandial 2-h glucose levels ≥ 11.1 mmol/L from an oral glucose tolerance test, or random glucose levels ≥ 11.1 mmol/L [21]. Self-reports, antihypertensive drug use, and mean systolic or mean diastolic blood pressure readings (≥140/90 mmHg) were used to diagnose hypertension.

### Statistical analysis

Sample weights, strata and primary sampling units were employed in all analyses to create a representative sample of the national population. Normally distributed continuous variables were presented as means (standard error, SE), while nonnormally distributed variables were shown as medians (interquartile range, IQR). Numbers (percentages) were used to describe categorical variables. The quartiles of RC were divided according to their distribution in the study population. One-way ANOVA or the Kruskal-Wallis test was applied to compare continuous variables with or without a normal distribution among the four groups, respectively. The χ^2^ test was applied to compare categorical variables.

The odds ratios (ORs) and 95% confidence intervals (95% CIs) of reduced kidney function or albuminuria in relation to RC were determined by multivariate logistic regression model. Three fitted models were applied. Model 1 was modified by sociodemographic factors, including age, sex, race, educational background, and the household income- to- poverty ratio. BMI and smoking status were further incorporated into model 2. Model 3 was further modified by diabetes, hypertension, coronary heart disease and lipid-lowering treatment. The associations of RC with eGFR or ACR were also examined by adjusting linear regression models with the above confounders. Employing a restricted cubic spline (RCS) regression model, the dose‒response associations of RC with eGFR or ACR were further analyzed. Nonlinearity tests were performed.

Sensitivity analysis was performed by treating RC as a binary variable based on the inflection point of RCS used to determine the association between RC and ACR. Stratified analysis was conducted by the parameters of age (< 40, 40–60, ≥ 60 years), gender (male or female), ethnicity (non-Hispanic white or other), BMI (< 25, 25–30, ≥ 30 kg/m^2^), diabetes (yes, no), hypertension (yes, no), eGFR (< 60, ≥ 60 ml/min/1.73 m^2^) and albuminuria (yes, no) (subgroups for *P* for interaction).

R 4.2.1, a software platform developed by The R Foundation (http://www.R-project.org), was employed for statistical analysis. Significance was established as *P* value < 0.05.

## Results

The participants in the final analysis had an average age of 43.18 years. 48.87% of the individuals were male, while 41.92% were of non-Hispanic white ethnicity. The mean RC was 0.61 mmol/L. In those with and without decreased eGFR or albuminuria, the average RC level was 0.70 mmol/L and 0.60 mmol/L, respectively. Table 1 illustrated the baseline characteristics of the recruited participants. Individuals with a higher RC tended to be older, male, frequent smokers, and less educated with a lower income. They were also more likely to be obese, diabetic, and hypertensive. Across the lowest to the highest quartiles of RC, the prevalence of decreased eGFR or albuminuria was 284 (6.62%), 316 (7.10%), 418 (9.61%), and 533 (12.30%), for a total of 1551 (10.98%) or 15.1 million people.

**Table 1:** The clinical and biochemical characteristics based on RC quartiles in the NHANES participants

Characteristics	Total sample (N = 14,210)	Remnant cholesterol (mmol/L)				*P* value
		Quartile 1 (N = 3520)	Quartile 2 (N = 3546)	Quartile 3 (N = 3510)	Quartile 4 (N = 3544)	
**RC (mmol/L)**	0.61 (0.00)	0.27 (0.00)	0.44 (0.00)	0.63 (0.00)	1.11 (0.01)	< 0.0001
**Age (years)**	43.18 (0.21)	39.62 (0.33)	42.60 (0.35)	44.21 (0.30)	46.34 (0.30)	< 0.0001
**Male, n (%)**	6900 (48.87)	1402 (40.03)	1672 (46.60)	1800 (51.94)	2026 (57.81)	< 0.0001
**Ethnicity, n (%)**						< 0.0001
Non-Hispanic White	5919 (41.92)	1318 (63.43)	1454 (67.22)	1501 (68.61)	1646 (71.50)	
Non-Hispanic Black	2948 (20.88)	1118 (17.84)	848 (12.71)	603 (8.84)	379 (5.77)	
Mexican American	2500 (17.71)	420 (6.60)	543 (7.62)	718 (9.55)	819 (10.21)	
Hispanic	1237 (8.76)	251 (4.66)	308 (5.17)	333 (5.57)	345 (5.24)	
Other Races	1516 (10.74)	413 (7.47)	393 (7.28)	355 (7.43)	355 (7.27)	
**Education, n (%)**						< 0.0001
< High school diploma	3209 (22.73)	590 (11.20)	760 (14.05)	871 (16.93)	988 (17.54)	
High school diploma	3161 (22.39)	727 (20.08)	808 (23.62)	794 (22.89)	832 (25.53)	
≥Some college	7750 (54.89)	2203 (68.72)	1978 (62.32)	1845 (60.18)	1724 (56.93)	
**Income-to-poverty, n (%)**						0.55
< 1	2884 (20.42)	672 (13.62)	767 (14.79)	693 (13.81)	752 (13.73)	
**Smoking, n (%)**						< 0.0001
never	7851 (55.6)	2286 (63.72)	2034 (56.17)	1864 (51.51)	1667 (46.62)	
former	3048 (21.59)	638 (19.76)	685 (21.07)	794 (23.57)	931 (27.84)	
now	3221 (22.81)	596 (16.51)	827 (22.76)	852 (24.93)	946 (25.54)	
**BMI (kg/m** ^**2**^ **), n (%)**						< 0.0001
< 25	4196 (29.72)	1645 (49.93)	1211 (36.29)	810 (25.02)	530 (13.97)	
25–30	4632 (32.8)	967 (27.61)	1185 (33.23)	1206 (33.75)	1274 (35.61)	
≥30	5292 (37.48)	908 (22.45)	1150 (30.47)	1494 (41.23)	1740 (50.41)	
**Diabetes, n (%)**	2180 (15.44)	259 (5.10)	443 (8.51)	606 (12.48)	872 (21.05)	< 0.0001
**Hypertension, n (%)**	4963 (35.15)	878 (20.63)	1137 (28.64)	1359 (35.44)	1589 (43.94)	< 0.0001
**Coronary heart disease, n (%)**	328 (2.32)	51 (1.24)	65 (1.75)	83 (2.16)	129 (3.47)	< 0.0001
**Lipid-lowering medicine, n (%)**	1933 (13.69)	285 (7.32)	424 (11.42)	537 (14.82)	687 (20.28)	< 0.0001
**Decreased eGFR ± albuminuria, n (%)**	1551 (10.98)	284 (6.62)	316 (7.10)	418 (9.61)	533 (12.30)	< 0.0001
**Decreased eGFR, n (%)**	382 (2.71)	56 (1.41)	70 (1.40)	109 (2.53)	147 (3.84)	< 0.0001
**eGFR (ml/min/1.73m** ^**2**^ **)**	98.85 (0.31)	103.32 (0.49)	98.89 (0.46)	97.81 (0.42)	95.34 (0.46)	< 0.0001
**Albuminuria, n (%)**	1296 (9.18)	248 (5.55)	270 (6.07)	340 (7.51)	438 (9.68)	< 0.0001
**log-transformed ACR**	0.86 (0.01)	0.84 (0.01)	0.83 (0.01)	0.86 (0.01)	0.92 (0.01)	< 0.0001

Data are presented as mean (SE) or n (%). SE, standard error; BMI, body mass index; eGFR, estimated glomerular filtration rate; ACR, urine albumin-to-creatinine ratio

According to the primary analysis, RC was not significantly associated with renal dysfunction, as indicated by declined eGFR and/or albuminuria (OR 1.24, 95% CI = 0.95, 1.61) after multivariate adjustment. Similarly, the secondary analyses showed no significant association between RC and an increased risk of kidney function decline or albuminuria. However, a markedly adverse relationship between RC and eGFR was observed according to the fully adjusted linear regression model (β -2.12, 95% CI: -3.04, -1.21) (Table 2).

**Table 2:** Association between RC and decreased eGFR or albuminuria

Variable	Remnant cholesterol (mmol/L)			
	Quartile 1	Quartile 2	Quartile 3	Quartile 4
**Primary outcome**				
**Decreased eGFR ± albuminuria —OR (95% CI)**				
unadjusted	ref	1.08 (0.84,1.39)	1.50 (1.17,1.91) ^**^	1.98 (1.58, 2.48) ^***^
model1	ref	0.97 (0.74,1.25)	1.31 (1.01,1.69) ^*^	1.66 (1.30, 2.14) ^***^
model2	ref	0.95 (0.73,1.23)	1.23 (0.95,1.60) ^*^	1.53 (1.18,1.98) ^***^
model3	ref	0.90 (0.69,1.18)	1.12 (0.86,1.46)	1.24 (0.95,1.61)
**Secondary outcome**				
**Decreased eGFR—OR (95%CI)**				
unadjusted	ref	0.99 (0.59,1.67)	1.82 (1.17,2.83) ^**^	2.80 (1.77,4.43) ^***^
model1	ref	0.81 (0.47,1.37)	1.33 (0.84,2.12)	1.98 (1.23,3.18) ^**^
model2	ref	0.78 (0.45,1.36)	1.24 (0.77,2.01)	1.78 (1.07,3.01) ^**^
model3	ref	0.71 (0.41,1.25)	1.10 (0.68,1.80)	1.43 (0.83,2.47)
**Albuminuria—OR (95%CI)**				
unadjusted	ref	1.10 (0.85,1.43)	1.38 (1.08,1.78) ^*^	1.82 (1.44, 2.31) ^***^
model1	ref	1.03 (0.79,1.35)	1.28 (0.99,1.67)	1.67 (1.28,2.17) ^***^
model2	ref	1.01 (0.77,1.31)	1.20 (0.92,1.56)	1.52 (1.16, 0.99) ^*^
model3	ref	0.97 (0.74,1.27)	1.10 (0.85,1.43)	1.22 (0.94,1.60)
**eGFR (ml/min/1.73m2) —**β** (95%CI)**				
unadjusted	ref	-4.43 (-5.54, -3.31) ^***^	-5.51 (-6.67, -4.35) ^***^	-7.98 (-9.24, -6.72) ^***^
model1	ref	-1.72 (-2.64, -0.80) ^***^	-1.35 (-2.23, -0.47) ^**^	-1.82 (-2.70, -0.94) ^***^
model2	ref	-1.83 (-2.75, -0.91) ^***^	-1.58 (-2.48, -0.67) ^***^	-2.13 (-3.04, -1.22) ^***^
model3	ref	-1.78 (-2.69, -0.88) ^***^	-1.52 (-2.42, -0.62) ^***^	-2.12 (-3.04, -1.21) ^***^
**log-transformed ACR—**β ** (95%CI)**				
unadjusted	ref	-0.01 (-0.03, 0.02)	0.02 (-0.00,0.05)	0.08 (0.05,0.10) ^***^
model1	ref	-0.01 (-0.03, 0.01)	0.02 (-0.01,0.04)	0.07 (0.04,0.09) ^***^
model2	ref	-0.01 (-0.04, 0.01)	0.01 (-0.01,0.04)	0.06 (0.04,0.09) ^***^
model3	ref	-0.02 (-0.04, 0.01)	0.00 (-0.02,0.02)	0.03 (0.01,0.06)

Model 1 was adjusted for age, sex, race or ethnicity (non-Hispanic white, non-Hispanic black, Mexican American, Hispanic, and other races), education (< high school, high school, and ≥ college) and family income-to-poverty (< 1 or ≥ 1). Model 2 was further adjusted for smoking (never, former, and current) and BMI (< 25, 25–30, and ≥ 30 kg/m^2^)

Model 3 was further adjusted for diabetes, hypertension, coronary heart disease, and lipid-lowering medication. ^*^P < 0.05, ^**^P < 0.05, ^***^P < 0.001 vs. ref

**Fig. 2:**
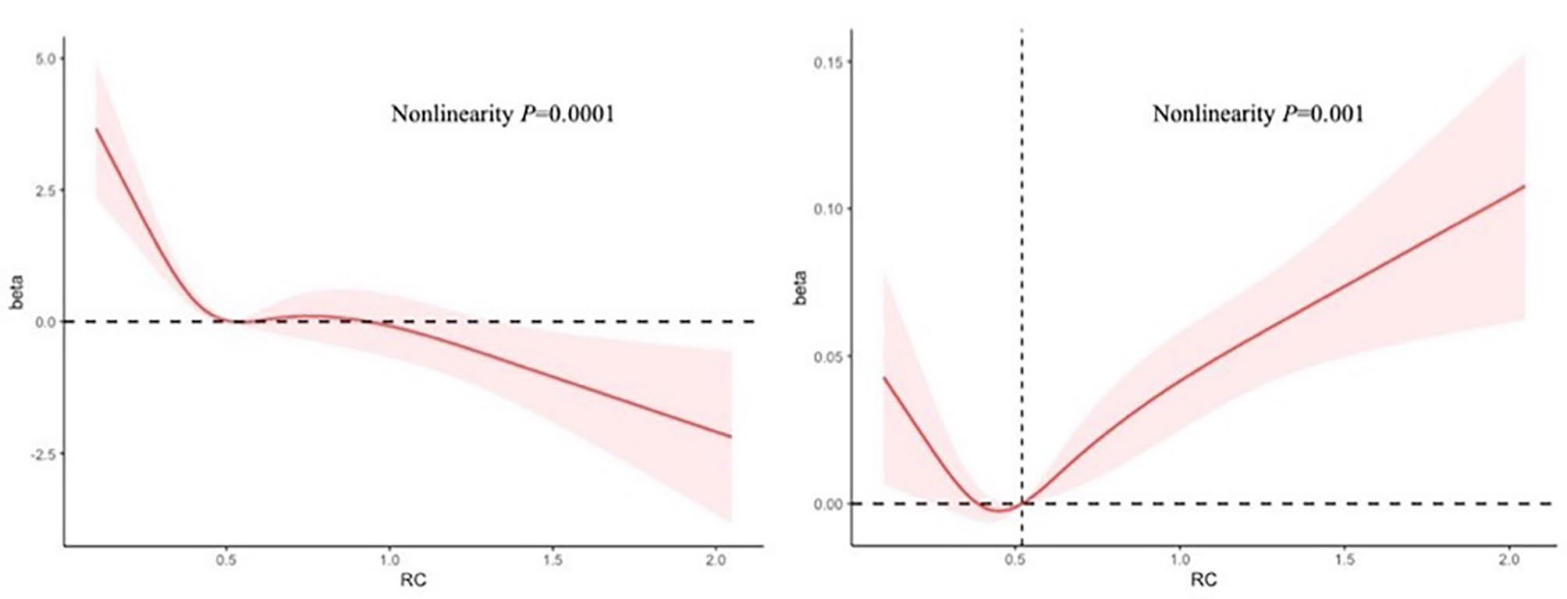
Dose-response relationship between RC and kidney function in NHANES participants. **A (Left)**: Association between RC and eGFR in a restricted cubic spline model. **B (Right)**: Association between RC and logACR in a restricted cubic spline model. Fully adjusted for age, sex, race/ethnicity, education, family income-to-poverty ratio, smoking, BMI, diabetes, hypertension, coronary heart disease, and lipid-lowering treatment. The shaded areas indicate the 95% CI

The dose‒response relationships of RC with eGFR and ACR were further investigated with an RCS regression model. Consistent with the findings of the secondary analyses, RC and eGFR levels were inversely associated in a linear fashion (nonlinear *P* = 0.0001; Fig. 2A). Moreover, there appeared to be a nonlinear relationship between RC and ACR, as evidenced by the J-shaped curve: the curve declined when RC increased to 0.52 mmol/L and then inclined with the elevation of RC levels (nonlinear *P* = 0.001; Fig. 2B).

In the sensitivity analyses, RC was treated as a binary variable. The results revealed that individuals with an RC ≥ 0.52 mmol/L were at increased risks of CKD (adjusted ΟR 1.25, 95% CI:1.04, 1.49) and decreased kidney function (adjusted ΟR 1.53, 95% CI:1.09, 2.14), but not albuminuria. According to the multivariate linear regression model, an RC ≥0.52 mmol/L correlated with a lower eGFR (adjusted β -0.82, 95% CI: -1.47, -0.17) and a slightly increased ACR (adjusted β 0.02, 95% CI: 0.01, 0.04) (Table 3).

**Table 3:** The logistic and linear regression analysis between RC and decreased eGFR or albuminuria

Exposure	Primary outcome	Secondary outcomes			
Remnant cholesterol	Decreased eGFR ± albuminuria OR (95%CI)	Decreased eGFR OR (95%CI)	eGFR β (95%CI)	Albuminuria OR (95%CI)	LogACR β (95%CI)
**< 0.52**	ref	ref	ref	ref	ref
≥**0.52**	1.25 (1.04, 1.49) *	1.53 (1.09,2.14) *	-0.82 (-1.47, -0.17) *	1.18 (0.97,1.42)	0.02 (0.01, 0.04) **

Fully adjusted for age, sex, race or ethnicity, education, family income-to-poverty ratio, smoking status, BMI, diabetes, hypertension, coronary heart disease, and lipid-lowering treatment. *P < 0.05, ** *P* < 0.01 vs. ref

**Fig. 3:**
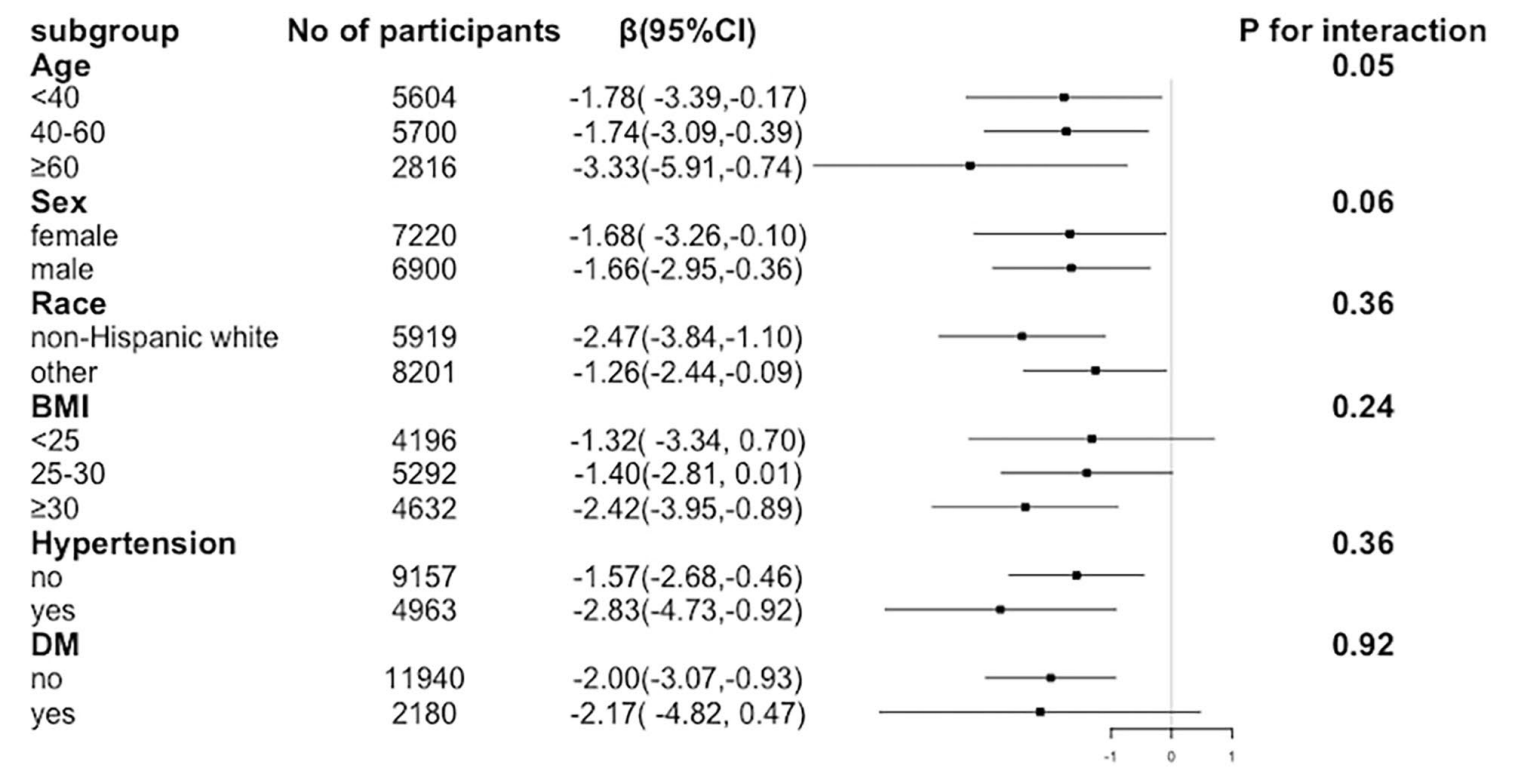
Effect size of remnant cholesterol on eGFR in subgroups. Adjusted for age, sex, race/ethnicity, education, family income-to-poverty ratio, smoking, BMI, diabetes, hypertension, coronary heart disease, and lipid-lowering treatment. The strata variable was not included in the model when stratifying by itself

The inverse association between RC and eGFR was consistent when participants were stratified by sex (male or female), age (< 40, 40–60, ≥ 60 years), ethnicity (non-Hispanic white or other), BMI (< 25, 25–30, ≥ 30 kg/m^2^), diabetes and hypertension; no significant interactions were detected after multiple tests (Fig. 3). Additionally, this correlation was unchanged in subgroups with or without decreased eGFR or albuminuria (Table S1). All these stratified analyses demonstrated a universally inverse relationship between RC and eGFR levels in the overall population. However, multiple covariates, including race, BMI, diabetes status, and hypertension, modified the effect of RC on the ACR (Fig. S1).

## Discussion

CKD patients with substantially high CVD risk commonly present with atherogenic dyslipidemia. Dysregulated metabolism in circulating TRLs plays a major role in CKD-associated lipid disorders [22]. Most cells can efficiently metabolize TG but not cholesterol. Therefore, it has been proposed that the detrimental element of TRLs is cholesterol rather than TG. In addition to traditional lipid measurements, RC, the cholesterol content of TRLs is independently associated with CVD risk [13, 23]. However, studies examining its association with renal insufficiency, specifically in the general population, have been scarce. This study examined the relationship between RC and impaired renal function or albuminuria in a large multiethnic cohort. The results showed an inversely dose-dependent relationship between RC and eGFR, independent of demographic and conventional cardiovascular risk factors. The validity of our conclusions was further supported by stratified and sensitivity analyses.

Several studies have shown increased levels of RC and its components in people with albuminuria, CKD, or end-stage renal disease (ESRD) [15, 24, 25]. For instance, RC levels were increased in CKD patients and positively correlated with CKD stage in a cross-sectional study involving 395 nondiabetic individuals [16]. Two studies conducted on Japanese patients with T2DM demonstrated that diabetic nephropathy progressed to overt proteinuria accompanied by elevated RC levels [15, 26]. In contrast, Ryzy et al. [27] observed no significant differences in VLDL or IDL-C between CKD patients and healthy individuals. In addition, a prospective cohort study involving 3,939 CKD patients revealed a nonindependent association between VLDL-C and worsening kidney function [10]. The differences in population characteristics, sample size, measurement techniques, and confounding factors in the analysis may explain the inconsistencies across these studies.

The negative association of RC with eGFR remained significant after controlling for all confounding variables, demonstrating an independent relationship between elevated RC and a lower eGFR. The European Atherosclerosis Society recommended that patients have desirable RC concentrations below 0.8 mmol/L in the fasting state to reduce CVD risk [28]. Specifically, this study showed an increased risk of CKD and decreased kidney function associated with RC > 0.52 mmol/L, suggesting that a lower cutoff of RC may be helpful for the early detection of individuals with impaired kidney function. The negative correlation between RC and eGFR in elderly patients was marginally strengthened in the subgroup analysis. Population-based studies have confirmed the substantial increase in CKD prevalence in the aging population, which is prone to dysregulated lipoprotein metabolism [29]. Dyslipidemia may increase CVD risk attributable to CKD in elderly patients. Interestingly, this study demonstrated a nonindependent relationship between RC and ACR, which was modified by several variables, particularly cardiovascular risk factors. Yan et al. [30] also observed that the association between RC and CKD risk was interacted by obesity, diabetes, and hypertension, indicating a complex relationship between RC and albuminuria. As obesity, diabetes, and hypertension are causative factors of albuminuria [31, 32], further investigation is needed to clarify the involvement of RC in albuminuria.

### Strengths and limitations of the Study

This study’s advantage is the calculation-based measurement of RC, which is reliable and commonly used in previous studies [33, 34]. While nuclear magnetic resonance (MR) or ultracentrifugation provides standardized measurements, these methods are time-consuming, expensive and radioactive, making them unsuitable for clinical practice. To our knowledge, the correlation between RC and kidney dysfunction in the general population was examined for the first time. Notably, the sample in this cohort was large enough to reduce the discrepancies caused by several variables, including demographic variables, cardiometabolic factors and measurement methods. In addition, there are several limitations to this study. Only associations, but not causality, can be established with this study design; therefore, prospective studies elucidating the causative role of RC in renal function deterioration are needed. Moreover, other potential confounders, such as dietary factors, genetic factors, and other chronic diseases that may affect RC levels, were not completely ruled out.

## Conclusions

This observational study of the general population demonstrated that increased RC was significantly associated with a lower eGFR. RC measurements may aid in the early detection of individuals with a lower kidney function. The causal relationship between RC and kidney outcomes needs to be further elucidated in future longitudinal studies.

### Electronic supplementary material

Below is the link to the electronic supplementary material.

**Additional File 1**: Table S1: Subgroup analyses examining the association of RC with eGFR. The analyses were fully adjusted for age, sex, race or ethnicity, education, family income-to-poverty ratio, smoking, BMI, diabetes, hypertension, coronary heart disease, and lipid-lowering treatment. Results showed that the negative correlation of RC with eGFR was independent of decreased kidney function or albuminuria

**Additional File 2**: Figure S1: Effect size of RC on log ACR in subgroups. The analyses were adjusted for age, sex, race or ethnicity, education, family income-to-poverty ratio, smoking, BMI, diabetes, hypertension, coronary heart disease, and lipid-lowering treatment. The strata variable was not included in the model when stratifying by itself. Multiple covariates, including race, BMI, diabetes, and hypertension, modified the effect of RC on ACR, according to the results

## Data Availability

The datasets supporting the conclusions of this article are available in the 2001–2018 continuous National Health and Nutrition Examination Survey, http://www.cdc.gov/nhanes/.

## Abbreviations

CKD: Chronic kidney disease
CVD: Cardiovascular disease
eGFR: Estimated Glomerular Filtration Rate
ACR: Albumin-to-creatine ratio
BMI: Body mass index
NHANES: National Health and Nutrition Examination Survey
RC: Remnant cholesterol
TG: Triglyceride
TRLs: TG-rich lipoproteins
VLDL-C: Very low-density lipoprotein-cholesterol
IDL-C: Intermediate-density lipoprotein cholesterol
LDL-C: Low-density lipoprotein cholesterol
HDL-C: High-density lipoprotein cholesterol
CKD-EPI: Chronic Kidney Disease Epidemiology Collaboration
CRIC: Chronic renal insufficiency cohort
ESRD: End-stage renal disease
Mn: Manganese
MR: Magnetic resonance
