# Supplementary material for: Association of remnant cholesterol with decreased kidney function or albuminuria: a population-based study in the U.S
Source: Lipids Health Dis. 2024 Jan 4;23:2. doi: 10.1186/s12944-023-01995-w (PMC10765762; doi:10.1186/s12944-023-01995-w)
Supplement: Supplementary file 1 — Additional File 1: Table S1: Subgroup analyses examining the association of RC with eGFR. The analyses were fully adjusted for age, sex, race or ethnicity, education, family income-to-poverty ratio, smoking, BMI, diabetes, hypertension, coronary heart disease, and lipid-lowering treatment. Results showed that the negative correlation of RC with eGFR was independent of decreased kidney function or albuminuria [file 12944_2023_1995_MOESM1_ESM.docx]

Supplementary material

Table S1. Subgroup analyses examining the association of remnant cholesterol with eGFR

| **Exposure** | **Kidney function** | |
| --- | --- | --- |
| **Remnant cholesterol** | **eGFR(ml/min/1.73m^2^)** | |
|  | **Multivariable-β(95% CI)** | ***P* for interaction** |
| **eGFR** |  | 0.37 |
| ≥60 | -1.63(-2.55, -0.72) |  |
| <60 | -3.18(-6.62,0.26) |  |
| **albuminuria** |  | 0.23 |
| no | -1.90(-2.97, -0.83) |  |
| yes | -2.12(-5.53, 1.29) |  |

fully adjusted for ﻿for age, sex, race/ethnicity, education, family income-to-poverty ratio, smoking, BMI, diabetes, hypertension, coronary heart disease, and lipid-lowering treatment.
