# Supplementary figures and images for: Association of remnant cholesterol with decreased kidney function or albuminuria: a population-based study in the U.S
Source: Lipids Health Dis. 2024 Jan 4;23:2. doi: 10.1186/s12944-023-01995-w (PMC10765762; doi:10.1186/s12944-023-01995-w)

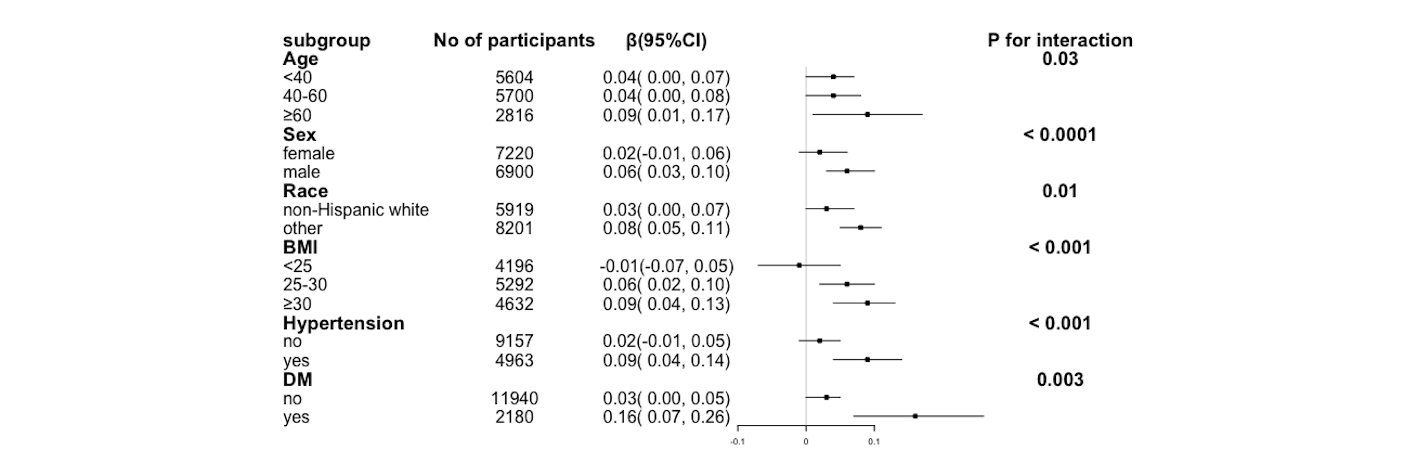

Supplement: Supplementary file 2 — Additional File 2: Figure S1: Effect size of RC on log ACR in subgroups. The analyses were adjusted for age, sex, race or ethnicity, education, family income-to-poverty ratio, smoking, BMI, diabetes, hypertension, coronary heart disease, and lipid-lowering treatment. The strata variable was not included in the model when stratifying by itself. Multiple covariates, including race, BMI, diabetes, and hypertension, modified the effect of RC on ACR, according to the results [file 12944_2023_1995_MOESM2_ESM.jpeg]
